# You may say I’m a dreamer

**DOI:** 10.1080/19336934.2022.2162593

**Published:** 2023-01-01

**Authors:** Howy Jacobs

**Affiliations:** Faculty of Medicine and Health Technology, Tampere University, Tampere, Finland; Department of Environment and Genetics, La Trobe University, Melbourne, Victoria, Australia

Many authors of well controlled and properly documented studies are disappointed when they receive editorial rejection letters, advising them to submit their manuscript to ’a more specialized journal’. Although sometimes this reflects the fact that the submission has gone to the wrong place, it is a phrase that is often used disingenuously. For, in the next paragraph, authors are typically invited to consider transferring their manuscript to the publisher’s own beta or gamma journal, which turns out to be even less specialized than the one from which it is being rejected.

The recommendation is thus revealed to be a thinly disguised judgement of the editor that there is only a low chance of the paper, once it has gone through peer-review, being frequently cited. This *might* be due to a perceived lack of conceptual novelty in the work, which could be a justifiable reason, at least in the case of the top-ranked broad-interest journals. There is, after all, a great deal of competition for space. But, more often, authors suspect that there are other reasons. These might include, for example, whether other papers of that author have been widely cited, which may not be the case for a relatively junior scientist, or one who has recently switched topics. Papers authored by a perceived leader of the field are much more likely to be cited, since other authors will trawl in the literature using that person’s name as a search term, or will consider their work more trustworthy than that of an unknown newcomer. Part of that is the well-known postcode effect: the fact that the work of authors located outside of major centres, both in terms of geography and scientific repute, are often and unfairly considered inferior to manuscripts emerging from a top institution. Finally, there are considerations relating to the actual subject of the work, which those working on *Drosophila* will surely recognize. The huge emphasis on medical or other societal relevance as a mark of citability means that work of basic biologists, and especially those using a non-mammalian model organism, tends to be considered less interesting and important than, say, equivalent work using a human cell-line, however non-physiological its properties.

In the end these boil down to the fact that there is often an explicit or implicit pressure on editors to maximize the short-term citability of accepted articles, which ultimately determines the journal’s impact factor (IF). Obsession with IF is not confined to scientists who believe that their job prospects are dependent on publishing in ‘high-impact’ journals, despite the progress that has been made by the DORA initiative (https://sfdora.org/). Editors are victims of this system as much as authors. And publishers too, since IF is not just the currency of scientific publication, but also equates to real money. If a journal’s IF falls below a certain threshold, its financial viability is threatened. Even if such commercial judgements might seem inappropriate in the realm of scholarship, a journal that loses money simply cannot survive for long. Even if the whole of scientific publishing were reorganized on non-profit lines, there are real costs that would still need to be covered, for the system to function.

As someone who has been involved, on the editorial side, with different kinds of scientific journals, I nevertheless strive to take a different position. To me, a scientifically sound finding is worthy of publication, and the criteria for acceptance are the same in any journal. If the controls have been properly implemented, the statistical analysis is robust and the authors’ interpretation is reasonable, then it deserves to be in the public domain. Even if it advances the field only modestly, has been executed in a location few could place on a world map, or is about *Drosophila* rather than the malaria parasite. The decision as to how significant it is in the history of the world need not be taken by an editor. Commentaries by peers and others should suffice. And even the opinion of students, both today and in the far future.

As current editor of *Fly* I am proud to be at the helm of a journal that loudly proclaims itself as a specialized journal: specialized in reporting work conducted on *Drosophila*, on winged hexapods in general, and on anything else in science that has been inspired by, or relates to, prior work thereon. Even if, within that ambit, are included neuroscience, developmental biology, ecology, evolution and many other ‘disciplines’ which together make it in some ways not specialized at all.

I look forward to a day when all academic journals – if journals, as such, even continue to exist – will be of this type: collections of articles that unify one ‘angle’, even though they are not the be all and end all of scientific knowledge even of the limited field for which they serve as a beacon. Publishing will still not be free of charge, and editors will still have to exercise judgements based on peer-review. But I hope and even expect that the era of high-end journals that decide what is impactful and what isn’t will eventually come to an end, simply because such judgements are manifestly arbitrary and unreliable, and in the end will not even be financially justifiable.

As for *Fly*, I hope we can continue to attract papers that are *interesting*. But that is something that the community should judge, not me.

As already implied, I suspect that we might one day have only ‘the peer-validated literature’: I can even hear a latter-day scientific John Lennon proclaiming ‘Imagine there’s no journals, it’s easy if you try’. But in such a world, I would hope there would still be room for overlapping research communities to gather together and promote the findings they regard as relevant and appealing. Until then, let’s continue to value our ‘more specialized journals’, as a counterweight to spurious judgements of what is or is not sexy science.

